# Emerging imaging methods to study whole-brain function in rodent models

**DOI:** 10.1038/s41398-021-01575-5

**Published:** 2021-09-04

**Authors:** Marija Markicevic, Iurii Savvateev, Christina Grimm, Valerio Zerbi

**Affiliations:** 1 Neural Control of Movement Lab, HEST, ETH Zürich, Zürich, Switzerland; 2 Neuroscience Center Zurich, University and ETH Zürich, Zürich, Switzerland; 3 Decision Neuroscience Lab, HEST, ETH Zürich, Zürich, Switzerland

**Keywords:** Neuroscience, Predictive markers

## Abstract

In the past decade, the idea that single populations of neurons support cognition and behavior has gradually given way to the realization that connectivity matters and that complex behavior results from interactions between remote yet anatomically connected areas that form specialized networks. In parallel, innovation in brain imaging techniques has led to the availability of a broad set of imaging tools to characterize the functional organization of complex networks. However, each of these tools poses significant technical challenges and faces limitations, which require careful consideration of their underlying anatomical, physiological, and physical specificity. In this review, we focus on emerging methods for measuring spontaneous or evoked activity in the brain. We discuss methods that can measure large-scale brain activity (directly or indirectly) with a relatively high temporal resolution, from milliseconds to seconds. We further focus on methods designed for studying the mammalian brain in preclinical models, specifically in mice and rats. This field has seen a great deal of innovation in recent years, facilitated by concomitant innovation in gene-editing techniques and the possibility of more invasive recordings. This review aims to give an overview of currently available preclinical imaging methods and an outlook on future developments. This information is suitable for educational purposes and for assisting scientists in choosing the appropriate method for their own research question.

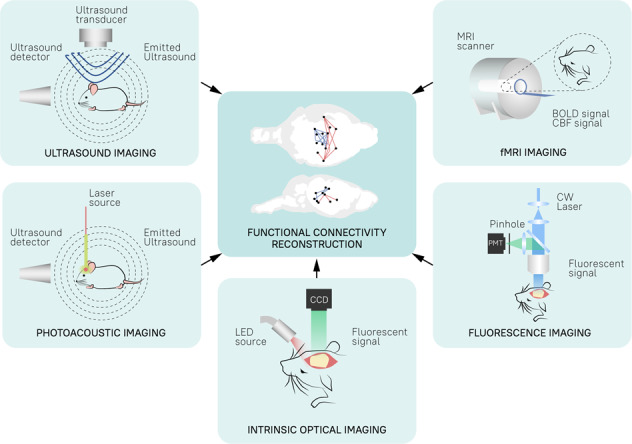

## Introduction

### Galaxies of thought, cognition, and movement

**Fig. 1:**
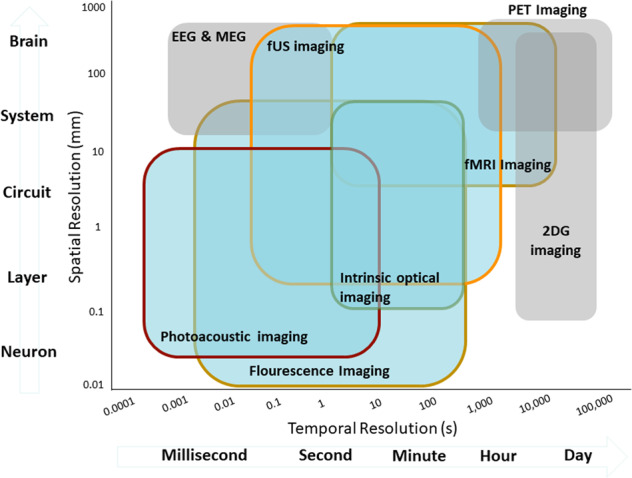
The spatiotemporal overview of imaging techniques used for studying rodent whole-brain function. Each colored box represents the approximate spatiotemporal scope of the labeled technique. Light blue colored boxes represent techniques covered in this review, while gray boxes techniques are not covered. *EEG* electroencephalography, *MEG* magnetoencephalography, *PET* positron emission tomography, *2-DG* 2-deoxyglucose, *fUS* functional ultrasound, *fMRI* functional magnetic resonance imaging.

The observation of natural phenomena is the basis of modern scientific thought, and a common approach to all scientific disciplines, from astronomy to neuroscience. Through observations, we can generate, confirm, extend or challenge theories and models of how nature works. And just as telescopes are the means of unlocking the secrets of outer space, our understanding of the brain depends on the methods we use to observe its constituent elements and study how they interact with each other, creating galaxies of thought, cognition and movement. While there is no single technique (yet) capable of observing all these phenomena, there are many technologies at our disposal to study brain activity across multiple temporal and spatial dimensions (Fig. 1).

The basic substrate used by the brain to transmit information is represented by electrical events called neuronal spikes and the release of chemical neurotransmitters in the synaptic terminals. Decades of (electro)physiological research facilitated by in vitro preparations, neuronal cell cultures or organoids, and in vivo recordings have advanced our understanding of the mechanisms that drive neurons to fire and transmit their signals through the network. Although neuronal rhythmicity has an essential role in facilitating information processing across spatial and temporal hierarchies in the brain [1], individual neural spikes per se are too weak to influence complex behavior (with notable exceptions) [2]. If our cognition really depended on individual spikes, we would deal with a poorly defined, high-dimensional system, not suitable for life. According to this view, correlates between the activity of a single neuron and a specific cognitive process provide a limited description of the causal relationship between brain activity and behavior [3]. Thus, it seems increasingly likely that the brain does not use actual spike coding but population—or neural ensembles—coding that unfolds on a limited, low-dimensional portion of the full neural space [4, 5]. As information flows through the brain, population activity is further integrated into large-scale networks via the connectome [6]. The result is that large numbers of brain regions are active during every aspect of cognition and behavior.

Since one of the more tractable goals of quantitative neuroscience is to develop predictive models that relate brain activity to behavior, observing activity, and dynamics in neural networks—possibly in multiple brain areas—can get us closer to this goal. To do that, scientists and engineers have developed an array of methods capable of looking at whole-brain activity from a zoomed-out perspective. In this review, we aim to provide the reader with an overview of the emerging methods for observing system and network-level brain function in rodents. Although this article is not designed to provide a full review of the literature, history, and physics behind each method, we distill the nature and the unique features of each technique and comment on their use and potential for future expansion and of course, their limitations. We wrote this article for scientists who want to expand their view on preclinical imaging methods, are looking for the appropriate method to address their research question, and for didactical purposes.

#### Functional MRI

Functional magnetic resonance imaging (fMRI) is one of the leading techniques to study whole-brain function in humans. Its first description dates back to the early 1990s, when Ogawa and colleagues [7] described the principles of blood oxygen level-dependent (BOLD) magnetic resonance imaging (MRI): local changes in the neuronal activity require a dynamic supply of oxygen and glucose, provided by a highly dense vascular system. More specifically, the process of neurovascular coupling, which entails the acute regulation of cerebral blood flow (CBF) via vasoactive molecules and neural messengers, ensures that this change in energetic demand is met (extensively reviewed in refs. [8–11]). To this day, much research effort is directed to the identification of cellular and molecular messengers that communicate neuronal activity to the vasculature, helping us understand cerebrovascular regulation and more accurately interpret observed fMRI signals [12–17]. Ultimately, regional alterations in CBF influence the ratio of oxygenated vs deoxygenated hemoglobin, whose distinct magnetic properties give rise to the BOLD signal. It is the paramagnetic properties of the deoxyhemoglobin that cause magnetic susceptibility inside blood vessels and surrounding tissue, thus affecting the magnetic field and the spin-spin relaxation time (T2/T2^*^).

MRI sequences that are sensitive to the T2^*^, such as gradient echo (GRE) echo-planar imaging (EPI), are often used for studying the fast dynamics of hemodynamic responses with a spatial resolution of ~1–3 mm and with a temporal resolution of ~1–3 s [18, 19]. In 1995, Biswal et al. [20] showed that also slow (<0.1 Hz), spontaneous fluctuations of the BOLD signal measured during rest periods (i.e., without the overt perceptual input or motor output typically present in traditional task-based fMRI studies) could be measured with a GRE-EPI sequence. These fluctuations form spatial patterns of correlated activity (i.e., networks) that unfold along with the long-range axonal connections of the brain, revealing its intrinsic functional architecture [21]. Since then, resting-state fMRI (rsfMRI) has become the method of choice to map regional interactions that occur in a resting or task-negative state across the whole brain in humans (with ~16.000 rsfMRI papers published in the past two decades; source: Pubmed, Jan 2021). To this day, GRE-EPI sequences with intrinsically high T2^*^ sensitivity, high temporal resolution, and signal stability are the most common choice for BOLD fMRI. It should be noted, though, that few technical caveats of the T2^*^ contrast remain [22]. In fact, GRE sequences are more frequently troubled by susceptibility and chemical shift artifacts given their high sensitivity to large vessels, potentially leading to overestimations of activated regions [22, 23]. However, there are other types of MRI sequences that can work around these problems. The most common are T2-weighted techniques such as EPI spin-echo sequences, which filter signals associated with larger veins and enhance contrast from small capillaries that are more likely to be closer to the site of neuronal activity [24]. This advantage comes at the expense of lower BOLD sensitivity and longer acquisition times [25, 26]. Given that different techniques of contrast, weighting are likely to involve different trade-offs between sensitivity and spatial specificity of the observed fMRI responses, making an informed decision regarding the choice of MRI pulse sequence to best fit one’s research question and study design is advisable.

##### Rodent functional MRI

Although the evidence provided by fMRI imaging in humans continues to teach us a lot about brain–behavior relationships, studying the biological underpinnings, which underlie large-scale networks and dynamics requires interventional and controlled experimental conditions only achievable in animal models. The past 10 years have seen a rapid increase in studies applying rsfMRI in rodent models. Early work mapped the spatial extent of resting-state networks in rats [27–30] and mice [31]. These findings have led to the realization that the rodent brain is organized in large-scale networks, the properties of which are similar to those reported in humans. This laid the foundation for studying the rodent brain using multiple approaches adapted from the human literature, such as independent component analysis [32–35], seed-based correlation [33, 36–39], dynamic functional connectivity analysis [40–42], and tools from graph theory [43, 44]. Other work focused on the relationship between network function and neuronal axonal connectivity, for example, by comparing rsfMRI data with the underlying anatomical connectivity from tracer injection experiments by the Allen Institute [45]. Thanks to this work, we have learned that high functional connectivity emerges predominantly between monosynaptically connected regions in the cortex, albeit this relation is not always present in subcortical regions like the thalamus [46]. In another study, Mills et al. [47] showed that, in addition to neuro-anatomical wiring, the genetic profiles of individual brain regions strongly contribute to functional connectivity, and that the variance of fMRI signals is best explained by a linear combination of axonal and gene expression data.

Rodent fMRI has also been used to better elucidate the mechanism of BOLD, for example, by combining it with direct measurement of neural and astrocytic activity [48–52]. Schlegel et al. [48] performed sensory-evoked (hind-paw stimulation) astrocytic and neuron-specific calcium recordings with simultaneous BOLD fMRI, and showed strong correlations between BOLD and calcium signals (both neuronal and astrocytic). Similarly, Tong and colleagues [50] revealed a strong coupling between the neuronal calcium signal and task-based and task-free BOLD responses.

Given that rodent and human fMRI share the same sequences for signal generation and use the same (pre)processing techniques, the technique holds great promise to understand the biological basis of human pathologies and link those with clinical outcomes in patients. Genetically modified (transgenic) animal models provide a crucial advantage in this undertaking. An extensive list of transgenic models has been investigated using rsfMRI to study numerous neuropsychological conditions, including Alzheimer’s disease [53–55], schizophrenia [56], pain [57], and autism [58–61]. For example, a recent study mapped the connectivity changes in subjects with autism-associated 16p11.2 deletion and in the mouse model with the same genetic mutation. Both groups displayed diminished functional connectivity in allegedly homologous brain networks [44]. In this context, fMRI represents a valuable tool for addressing the growing need to formally identify common brain circuits between rodents and humans to determine the scope and limits of rodent translational models [62, 63]. One caveat is that rodent fMRI is usually carried out in the anesthetized state to minimize head-motion during scanning, and only a handful of labs are acquiring fMRI data in awake animals [15, 64, 65]. The choice of anesthetic introduces confounds in fMRI measurements and is certainly a limitation for translating findings to humans [38, 39].

##### New avenues

New acquisition sequences to assess brain function with MRI are emerging at an ever-increasing rate. A family of MRI methods assesses brain activity in rodents (other than BOLD) by measuring cerebral blood volume (CBV) and CBF, usually referred to as perfusion MRI. CBV and/or CBF are often measured by injecting a paramagnetic contrast agent (CA) into the bloodstream [66]. The CA’s passage causes transient magnetic field inhomogeneities and introduces phase distortion of the water proton spins resulting in changes in T1, T2, or T2^*^ relaxation times, which can be captured with different MRI sequences. Commonly used CAs are paramagnetic gadolinium chelates, which increase T1, and superparamagnetic iron oxide nanoparticles, which decrease T2/T2^*^ (for a thorough review of techniques of perfusion MRI and comparison of CA see [66–68]). CBV-weighted fMRI in small animals has some advantages over BOLD measurements, including higher signal-to-noise ratio (SNR) and reduced susceptibility artifact. Furthermore, CBV represents a direct and easily interpretable component of the neurovascular cascade compared with the BOLD signal [66]. CBV-fMRI in combination with chemogenetic or optogenetic neuromodulation has been used to study the influence of serotonergic transmission on brain function. Giorgi et al. [69] measured the effects of pharmacological and chemogenetic serotonin modulation on whole-brain CBV. Their results indicate that serotonin modulation changes the CBV in multiple primary target regions of serotonin encompassing corticohippocampal and ventrostriatal areas. A similar study from Grandjean et al. [70] showed that optogenetic activation of the dorsal raphe nucleus resulted in a CBV decrease in primary target regions of serotonin. In addition, inducing acute stress by forced immobilization prior to the MRI also decreased the CBV in the same dorsal raphe’s primary target regions.

Other MRI sequences have been developed to achieve faster recordings, artifact-free images, or increase the specificity of MRI responses in relation to the underlying neural signals. Compared with conventional fMRI sequences, ultrafast fMRI sequences aim to shorten the repetition time (TR), which is the time from the application of a radiofrequency excitation pulse to the application of the next pulse. This can be achieved in a number of ways. One approach is to use simultaneous multi-slice imaging. Recently, Lee et al. [71] developed a sequence for rodent fMRI that can encode multiple slices simultaneously by using slice-select gradient blips. Blips impose different amounts of linear phase for different slices; thanks to an extended field of view (FOV), each slice is shifted towards a different and non-overlapping portion of the FOV, thus speeding up the acquisition by a factor of 4, while keeping a similar SNR to conventional EPI sequences.

Being able to use high spatiotemporal resolution is also critical to discern the direction of information flow using the onset times of fMRI responses. For example, Jung and colleagues [72] applied a GRE-EPI sequence at an ultra-high magnetic field (15.2 T) with a temporal resolution of 250 ms and spatial resolution of 156 × 156 × 500 μm^3^ during either electrical paw stimulation or optogenetics stimulation of the motor cortex. Their results showed that the order of onset times varies between regions and active layers and coincides with their known sequence of neural activation. This work provided further evidence that ultra-high resolution BOLD fMRI can be useful to identify bottom–up and top–down processes between cortico-cortical and cortico-thalamic regions and to assess the direction of information flow.

Images generated by conventional fMRI sequences in rodents suffer from high sensitivity to magnetic susceptibility artifacts due to the high field of scanners and the relatively long echo time required to generate the BOLD contrast. To solve this problem, MacKinnon and colleagues combined a zero-time echo (ZTE) pulse sequence with iron oxide nanoparticles to acquire CBV. The ZTE sequence is characterized by a very short echo time, which means that signal acquisition occurs immediately after the radiofrequency pulse, preventing signal decay. At the same time, iron oxide nanoparticles shorten the T1 relaxation time, resulting in the detection of CBV-weighted functional activations in the brain even with a low echo time. This allows for a threefold increase in the magnitude of the SNR, along with a reduction in susceptibility artifacts and acoustic noise [73].

New MRI sequences have also been developed to measure brain activity differently from the hemodynamic response. One example is given by diffusion functional imaging (dfMRI). In dfMRI, a spin-echo echo-planar (SE-EPI) sequence is combined with an isotropic diffusion encoding (IDE) gradient, to impart isotropic diffusion-weighting contrast in the acquired signal. This makes dfMRI sensitive to rapid changes in three-dimensional tissue boundaries induced by neuronal activation [74, 75]. Evidence from intrinsic optical signals (IOS) studies suggests a strong coupling between neural activity and microscopic (sub)cellular morphological changes [76]. Therefore, dfMRI was developed to detect changes in water diffusion properties related to “cell swelling” and coupled to neuronal activity rather than hemodynamic responses [74, 77]. Nunes et al. [78] were the first to investigate in greater depth the mechanism underlying neuromorphological coupling by developing an ultrafast line-scanning dfMRI SE-EPI sequence with a time resolution of 100 ms, which enabled the detection of rapid diffusion dynamics. Upon forepaw stimulation, they detected in the rat somatosensory cortex that the dfMRI signal contains two different components: a fast-onset component that is insensitive to vascular change, followed by a slower component sensitive to vascular change. Independent IOS of optogenetically stimulated brain slices confirmed the close similarity between fast IOS and the fast-onset dfMRI component, thus suggesting further evidence of neuromorphological coupling. Moreover, in human studies, dfMRI showed higher spatial accuracy at activation mapping compared with classic functional MRI approaches [79] [80]. Nunes and colleagues [81] applied dfMRI in rodent fMRI, and tested the specificity of dfMRI by mapping whole-brain responses upon hind-paw stimulation with voxel resolution. Their results indicated that the dfMRI signal exhibits layer specificity and is spatially overlapping with the underlying neural activity within the thalamocortical pathway.

Another family of methods that have recently been developed to evaluate neural activity is called molecular fMRI. Molecular fMRI monitors brain activity through the use of chemical or genetically encoded probes, i.e., MRI molecular imaging agents, which are designed to bind to specific molecular and cellular targets in the brain, analogous to fluorescent dyes for optical imaging [82–84]. These MRI molecular imaging agents work by interacting with water molecules to alter T1 and T2 relaxation times, or in some cases by incorporating nuclei that can be probed using radio frequencies distinct from those used to measure water protons [83, 85]. Thus, molecular fMRI readout reflects the distinct molecular hallmarks of neural activity, rather than hemodynamic coupling that underlies BOLD fMRI. The first molecular fMRI study to combine molecular specificity and spatial coverage using a neurotransmitter sensor detectable by MRI assessed dopamine signaling. They injected MRI CA sensitive to dopamine into the rat nucleus accumbens (NAc) and measured changes in dopamine concentration in NAc and caudate-putamen (CPu) upon electrical stimulation of the medial forebrain bundle in the lateral hypothalamus [86]. Recently, this approach was combined with BOLD fMRI [87], where simultaneous functional BOLD and molecular imaging responses were recorded throughout the rat brain using multi-gradient echo MRI pulse sequence, during electrical stimulation of the hypothalamus. Results indicated that phasic dopamine release in the NAc and medial CPu alters the duration, but not the magnitude, of the stimulus responses across the striatum via postsynaptic effects that vary across subregions, and that dopamine causally modulates BOLD fMRI responses in the distal cortical regions.

Another method that allows quantitative and non-invasive assessment of cerebral metabolism during brain activity is functional magnetic resonance spectroscopy (fMRS). The goal of fMRS is to obtain precise quantitative in vivo measurements of various metabolic concentration changes during brain activity. Although broadly used in human brain studies, its application in rodents is still limited mainly due to low SNR, low (~4 sec) temporal resolution, and anesthesia confounds [88, 89]. A recent review gives a detailed overview of the methodological aspects and translational prospects of fMRS in rodents [88].

#### Ultrasound imaging

Ultrasound imaging is a widely used diagnostic technique in medicine that is based on the principle of the emission of ultrasonic waves (from 20 KHz to ~15 MHz) and the transmission of echoes. Using the speed of sound and the time of each echo’s return, an ultrasound system calculates the distance from the transducer to the tissue boundary and then uses this information to generate images of tissues and organs [90]. Ultrasound systems can also be tuned to assess blood flow using the Doppler effect. The principle of Doppler ultrasound consists of detecting the movement of red blood cells by repeating pulsed emissions and studying the temporal variations of subsequent backscattered signals [91]. In clinics, Doppler ultrasound is the most commonly used technique to study blood circulation in the heart, arteries, limbs, kidneys, and liver. However, for the brain, the application of transcranial Doppler (TCD) ultrasound is limited owing to strong attenuation of the ultrasound beam by the skull, and its only clinical use is to diagnose cerebrovascular pathologies in newborns through the fontanel (for a review of TCD applications see [92]).

##### Functional ultrasound imaging in rodents

Until recently, little work has been done in preclinical rodent neuroscience using TCD. Although partial skull removal could resolve scattering problems, conventional ultrasound still suffers from low sensitivity, which limits its application to image blood volume or flows in major cerebral arteries. However, the development of new concepts and technologies, such as ultrafast ultrasound and the use of plane-wave illumination as opposed to focused beam scanning, have enabled the use of ultrasound in basic neuroscience research [93]. Thanks to new scanners capable of acquiring images at a very high frame rate (~20 kHz), ultrafast ultrasound can boost the power Doppler SNR over 50-fold, without the need for CAs [90]. This increased sensitivity allows mapping of blood flow changes in small arterioles (up to 1 mm/s) that are related to small and transient changes in neuronal activity, laying the foundation for functional ultrasound (fUS) imaging (for a review on the techniques and physics of this technology see [90] and [93]).

The first application of fUS imaging in translational neuroscience appeared in 2011, when Macé and colleagues [94] showed the activation of the barrel cortex following whisker stimulation in anesthetized rats with high spatiotemporal detail. Furthermore, the authors measured the spatiotemporal dynamics of epileptiform seizures, showing cortical spreading depression propagating throughout the entire brain. Since then, more groups have started to use fUS as a tool to record whole-brain activity in many behavioral and cognitive tasks, such as forepaw electrical stimulation in rats [95], or at rest [96, 97]. In most cases, large cranial windows or skull thinning procedures were used for stable chronic imaging of deep brain structures. However, in 2017 Tiran et al. [98] showed that the whole-brain vasculature could be imaged through the skull and skin in awake and freely moving mice, whereas young rats can be imaged up to 35 days of age without prominent reductions in image quality. A year later, Macé et al. [99] used fUS to map the brain areas activated during optokinetic reflex in awake mice and functionally dissect the regions whose activity depended on the reflex’s motor output. To date, new strategies can further increase the resolution of acquired images while maintaining rapid acquisition, for example, using microbubble CAs and time tracking of microbubble positions [100].

In general, brain imaging in awake and behaving animals confers an advantage to fUS over fMRI. Furthermore, fUS combines whole-brain reading with a relatively high spatial resolution (100 × 100 × 300 µm) but with higher temporal resolution and low operating and maintenance costs. Although the skull remains an obstacle in fUS imaging for ultrasound wave propagation, the use of CAs [101] or a surgical procedure to produce a craniotomy or thinned skull window can solve this problem. Thanks to recent developments in injectable ultrasound contrast media or ultrafast high SNR sequences, an expansion of preclinical fUS applications in neuroscience is expected in the near future.

#### Fluorescence imaging

There are several optical imaging techniques that measure the activity of single neurons or neural groups, based on voltage or calcium dyes or genetically encoded probes. More recently, the field has seen strong development of methods that increase the visual field and allow large-scale measurements of neural activity. The basic principle common to these techniques lies in the light emission of specific chemical compounds named fluorophores. The fluorophore absorbs light of a specific wavelength that brings it from a ground state to an excited state. When the fluorophore relaxes back to the ground state, in a process named luminescence, it emits light at a specific wavelength and energy. The light emitted during the luminescence—the fluorescence signal—is then captured by the adjacent optical system. The two key factors defining a fluorescent imaging technique are (i) the type of fluorophore used [102, 103] and (ii) the design of the optical system [104]. In the following sections, we will address both factors with respect to in vivo functional brain imaging in rodents.

##### Fluorophores

Fluorophores can change their fluorescent properties, such as the wavelength or the intensity of the fluorescent signal, when involved in a specific physiological process, e.g., the firing of an action potential. The two most popular families of fluorophores used for in vivo brain imaging are (i) calcium and (ii) voltage indicators. Calcium indicators measure changes in intracellular calcium ion concentration, whereas voltage indicators assess alterations in the membrane potential.

Both calcium and voltage indicators can be further divided into two main groups: organic (e.g., chemical) [105–107] and genetically encoded [107, 108]. Organic calcium (e.g., fluo-4) or voltage (e.g., ANINNE-6) indicators are synthesized organic molecules that are delivered into a target cell via bulk loading or cell microinjections [105, 107, 109, 110]. In contrast, genetically encoded calcium (e.g., GCaMPs) or voltage (e.g., ASAPs) indicators are expressed directly by the target cell. The incorporation of the indicator genes is achieved by using transgenic animals and/or virus vectors designed to express the genetic material under the control of a tissue-specific promoter [107–109, 111]. The cell type-specific expression ensured by a tissue-specific promoter makes genetically encoded calcium indicators (e.g., GCaMPs) the most popular choice for in vivo brain fluorescent imaging in rodents.

Voltage indicators are sensitive to subthreshold membrane voltage dynamics and have a higher temporal resolution in comparison with the calcium indicators [107, 112] Nevertheless, their use for widefield in vivo brain imaging in rodents is hampered by the intrinsic low SNR [109] and by the technical challenge of achieving a precise localization of the voltage indicators in the cell membrane [107, 112]. Therefore, while recognizing recent developments [107, 112] and the potential of multiarea voltage imaging [112], we will focus our review on calcium indicators.

##### Optical designs

There are several optical systems currently available for detecting the fluorescent signal emitted by a fluorophore. Each of these can be used to maximize certain image parameters such as acquisition speed, spatial resolution, FOV, and dimensionality (e.g., 2D slice or 3D image) [104]. In the next sections, we focus on the most common fluorescence imaging procedures used for in vivo brain imaging in rodents. For each method, we explain the basic principles of the optical design and review work that demonstrates its application in studying brain function with a large FOV.

##### Wide-field fluorescence imaging

**Fig. 2:**
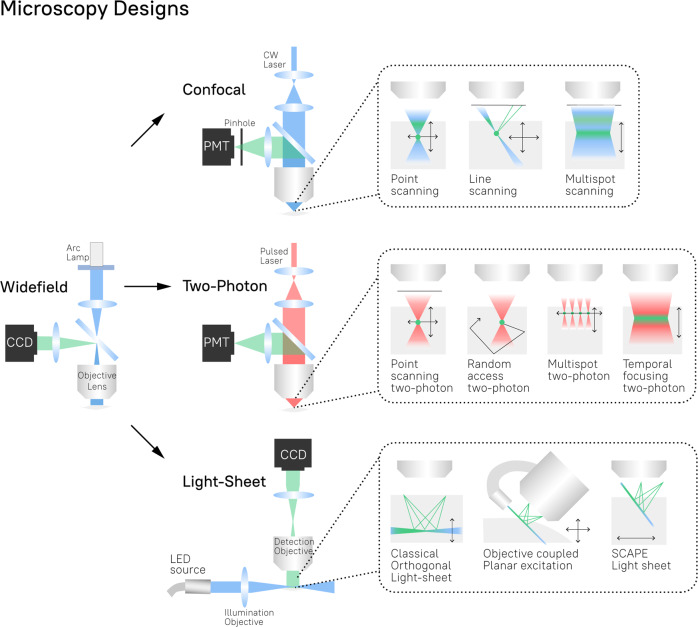
Microscopy designs. The main engineering components and operational modes of widefield, confocal, two-photon, and light-sheet microscopes. *LED* light-emitting diode, *CCD* charge-coupled device. Wide-field microscopes use arc lamps or LEDs (not shown) to produce a beam of light at a specific wavelength. In the case of Arc lamps filters (horizontal blue bulk) are used to select a specific wavelength from a continuous spectrum. The illumination light irradiates the entire specimen through the objective lens causing the excitation of fluorophores. The fluorescent signal emitted during the luminescence process is collected by the same objective lens and reflected by the dichroic mirror (deflected blue bulk) toward the CCD camera. Confocal microscopes use the same optical system as a wide-field microscope, with a few differences: (i) lasers are used as a light source, (ii) a “pinhole” is used to cut off the light outside of the focal plane, and (iii) a photo-multiplier tube (PMT) is used for image acquisition. Continuous-wave lasers (CW laser) are typically used in confocal designs ensuring the stable amplitude and wavelength of the illumination light. Depending on the operational mode the illumination is focused on the specific point (point-scanning), line (line-scanning), or multiple points (multisport scanning) inside a specimen. The black arrows feature possible scanning directions. The emitted fluorescent light is passed through the pinhole and ultimately collected by the PMT, which enhances the amplitude of the impingement light. Note that scanning mechanisms (XY) are not shown and that the confocal pinhole would need to be placed in the descanned pathway. When featured, XY scanner (e.g., galvo mirror scanner) is located on the detection pathway between the objective and dichroic mirror. Z-steppers or tunable acoustic gradient (TAG) lens for z-scanning is also not shown. When depicted, they are located at the back focal aperture of the objective. Multiphoton microscopes are represented by the example of the two-photon design. It utilizes the same components as the confocal, but with two principal differences: (i) a pulsed laser is used, (ii) a “pinhole” is not required. Two-photon microscopy is based on the two-photon excitation process: a fluorophore simultaneously absorbs two photons that together bring sufficient energy to cause the fluorophore excitation. Subsequent relaxation of the excited fluorophore back to the ground state is accompanied by the fluorescence emission. For the practical realization of the simultaneous absorption of two photons, a pulsed laser produces a beam of photons with the energy tuned for the two-photon excitation process of the targeted fluorophore: ½ of the excitation energy per photon. Since only the fluorophores at the focal plane can simultaneously absorb two photons, a pinhole is not used. Two-photon microscopes can be used in various scanning modes. Point-scanning mode refers to the illumination of a single point per scanning session. Random access is the point-scanning mode that is used for a set of predefined locations in the sample. Therefore, the random access technique does not image the whole specimen, but rather the part sufficient for the analysis. During a multispot session, multiple points are illuminated in parallel during the same scanning session. Finally, during the temporal focusing, the laser beam impinges on the diffraction grid, producing several beams that are further guided by the optical system to constructively interfere in the focal plane located at the specimen. Thus, the two-photon temporal focusing mode leads to the activation of a single plane. Light-sheet microscopes typically use an LED (or several) to illuminate the specimen from the side. Such an illumination process is called “Oblique Illumination”. The emitted fluorescent signal is captured by the objective lens and further transmitted to the CCD camera. Classical orthogonal light-sheet microscopy uses two orthogonal objectives: the first is for illumination and the second is for detection. This design requires a highly constrained sample geometry and either physical sample translation for 3D imaging or complex synchronization of illumination and detection planes. Both constraints limit the acquisition speed of classical orthogonal light-sheet microscopy. In the objective coupled planar excitation (OCPE) design the position of the illumination and detection objectives are mechanically coupled. Despite the ensured alignment of the illumination and detection planes, OCPE still requires mechanical movement of the coupled objectives to perform scanning. Finally, SCAPE microscopy acquires images using an angled, swept light sheet in a single objective. SCAPE permits three-dimensional imaging of intact samples at rates exceeding 20 volumes per second. [140, 200–204].

Wide-field fluorescence imaging (WF) refers to a procedure in which a whole specimen (e.g., the targeted brain regions or the entire cortex) is illuminated and the emitted fluorescence is recorded by a joined optical system (Fig. 2). In WF, it is essential to guarantee the passage of light with minimal dispersion and absorption; for this reason, WF is often combined with a procedure to replace the skull with a glass cranial window [113, 114]. For example, Kim et al. [114] measured GCaMP fluorescent signals with single-neuron resolution throughout the entire dorsal cortex in awake, head-fixed mice. However, WF can also be conducted through the skull by removing only the scalp. This severely limits the precision and resolution of the images but also minimizes invasiveness. Using WF imaging of GCaMP through the intact skull, Peters and colleagues [115] functionally mapped activity across the entire dorsal cortex in awake head-fixed mice, with a spatial resolution of 20 µm.

Animal experiments with head restraints impose a fundamental technical limit on the behavior that can be studied. To overcome this limitation, WF micro-endoscopes and optical fibers have been designed to visualize cortical and subcortical activity in awake and freely moving animals [116–120]. De Groot et al. [120] integrated two micro-endoscopes for simultaneous recording from distant brain regions, an inertial measurement unit for movement monitoring, and an LED driver for optogenetic stimulation in a single device named the “NINscope”. In one of their demonstrations, NINscopes were used to record the fluorescence of GCaMPs from the cortex and cerebellum and study the generation of movement upon cerebellar optogenetic stimulation in freely moving mice.

Despite the great potential of WF for in vivo brain imaging, its optical design suffers from a strong susceptibility to background fluorescence caused by the signal coming out of the focal plane. This places some fundamental constraints on WF applications. First, the background fluorescence decreases the SNR, which limits the spatial resolution and does not allow scanning subcellular structures (e.g., synaptic boutons). Second, as the depth of focus is determined by fixed parameters of the optics, there is a greater blur in the image when targeting deeper structures owing to the background signal from the regions above. This currently limits the depth of WF imaging to the superficial layers of the cortex. Finally, the continuous excitation of fluorophores from outside the focal plane can lead to an increase in phototoxicity and the production of reactive oxygen species (ROS), and photobleaching, which results in the inactivation of the fluorophore.

##### Confocal fluorescence imaging

Confocal microscopes use the same optical scheme as WF but with two additional features in order to increase the SNR. First, confocal microscopes utilize a tiny diaphragm, named a “pinhole”, to remove the signal coming out of the focal plane. Second, confocal microscopes use lasers as a light source instead of LEDs or arc lamps. This increases the focus of the illumination beam on the targeted region, whereas minimizing out-of-focus illumination. To achieve a large FOV, a complete 2D or 3D image of a sample is usually acquired by moving the illumination spot across the sample [104, 109, 121]. Confocal fluorescence imaging was used by Yoshida et al. [122] to measure GCaMP6 fluorescence from individual axonal boutons in behaving head-restrained mice. By using a multispot confocal design, they could image for the first time a relatively large FOV of 1 mm^2^. This allowed the study of long-range projecting axons from the thalamus to the primary motor cortex (M1) in layer 1. In order to circumvent the need for a head-fixation constraint, Dussaux and colleagues developed a fibrescope adaptation of the confocal design, which allows a FOV of 230 µm with single-cell spatial resolution. This design was used to study changes in the velocity of red blood cells in cortical microvessels in freely behaving mice compared with anesthetized animals [123]. Nevertheless, in the confocal design, the photons out of the focal plane still contribute to phototoxicity and photobleaching [124]. Another drawback is related to tissue scattering, which limits the depth of focus to a maximum of 100–200 µm [122, 123].

##### Multiphoton fluorescence imaging

Multiphoton microscopes use two or three photons to provide the energy needed to excite a fluorophore. The use of multiple photons means that the individual energy of each photon will be lower than that of a single excitation photon. Therefore, the corresponding wavelengths are shifted in the red/infrared part of the light spectrum, which is less scattered by brain tissues than other wavelengths. This gives multiphoton fluorescence imaging a greater depth of penetration than one-photon modes (e.g., WF, confocal). Also, owing to the greater focus of the laser beam, the fluorophores outside the focal plane do not absorb two photons (or three by three photons) simultaneously and are not excited. Therefore, the use of multiple photons ensures that the fluorophores are activated only in the focal plane, thus reducing background fluorescence and phototoxicity [124, 125].

Sofroniew et al. [126] used the two-photon design to simultaneously record GCaMPs signals from a circular FOV with a 5 mm radius, reaching depths of up to 1 mm. This was used to simultaneously monitor the activity from somatosensory, parietal, and motor cortical areas in head-fixed, behaving mice. At the same time, the high spatial resolution permitted the visualization of calcium signals from individual spines. In order to further increase the FOV, Yang et al. [127] introduced the “MATRIEX” design for multiarea two-photon imaging. MATRIEX uses multiple water-immersed miniature objectives, each having its own focal plane. Importantly, the images from these objectives can be simultaneously observed through one low-magnification dry objective. The use of several miniature objectives permitted not only simultaneous imaging from distant brain areas but also the ability to adjust the imaging depth of each region independently. This was used to simultaneously record GCaMP fluorescence from multiple brain regions distributed across an area up to 12 mm^2^ and located at different depths: primary visual cortex (V1), the primary motor cortex (M1), and CA1 region of the hippocampus. Wagner et al. [128] used two independent two-photon systems to image calcium activity from L5 of the premotor cortex and cerebellum granule cells (GrCs) with single-cell spatial resolution. The experiment was conducted on awake head-restrained mice and allowed the researchers to examine the activity patterns in L5 and GrCs during a motor learning task. In order to use multiphoton imaging in freely moving animals, miniature versions of two-photon microscopes were recently developed [129–131]. Zong et al. [131] used mini two-photon microscopes for calcium imaging in V1 in freely moving mice while keeping the spatial resolution at the level of individual spines, which is comparable to the conventional design. More recently, Klioutchnikov et al. [132] adopted a three-photon design for a head-mounted version and performed cortical imaging to a depth of 1.1 mm in freely moving rats with a spatial resolution of a few micrometers, enabling imaging of calcium signals from single soma and dendrites.

Despite these technical advances, both conventional multiphoton and confocal methods are point-scanning techniques. Therefore, they have a speed acquisition limit, which is set by the fluorescence lifetime of the fluorophore and the pulse of the laser. In other words, one can proceed with the next scanning point of the specimen only after the signal from the previous point is fully acquired. This limitation could be overcome by performing multiple scans in parallel, therefore, performing multispot multiphoton imaging (see Fig. 2 for details) [133, 134]. However, the parallel scanning of multiple spots requires spreading the laser beam to excite multiple spots in parallel, which reduces the excitation intensity at each individual spot, potentially compromising the fluorophore excitation. This constraint cannot be simply overcome by increasing the laser power owing to excessive heating of the tissue. An increase in the excitation intensity in the multispot two-photon design can be achieved by combining two-photon imaging with light-sheet microscopy [135, 136]. The light-sheet microscopy approach uses a side illumination that increases the chance of a photon being absorbed by the fluorophore in the desired plane (e.g., increased photon yield) while reducing photobleaching and phototoxicity, as it uses less laser power in comparison to confocal and multiphoton approaches. Photon light-sheet fluorescent calcium imaging has been used for in vivo functional imaging in awake head-restrained mice. However, it still has limited FOV (340 × 650 µm) and depth (135 µm), which restricted the analysis to only the motor cortex [137]. In vivo two-photon light-sheet imaging [135, 136, 138, 139] has not yet been applied for wide-field imaging in mice or rats. However, taking into account the rapid technological advances in multiphoton imaging, we anticipate that the combination of a two-photon setup and light-sheet microscopy will soon be implemented to further increase the FOV of functional fluorescent imaging.

#### Intrinsic optical imaging

Despite being one of the most widely used methods for in vivo wide-field fluorescent brain imaging, the use of artificial fluorophores (e.g., GCaMPs) is inevitably linked with either invasive interventions such as microinjections or with genetically modified organisms [109]. This impedes the application of these methods to other experimental animals (e.g., non-human primates) and humans. Intrinsic optical imaging (IOI) [106, 140] relies on the difference in light absorption between oxygenated and deoxygenated hemoglobin and provides a non-invasive alternative to fluorescent imaging. In brief, oxygenated hemoglobin (HbO_2_) has lower absorption at 630 nm in comparison with deoxygenated hemoglobin (HbR). This decreased absorption leads to higher reflectance, which can be detected by an optical system. The opposite effect occurs at 480 nm, where HbO_2_ has higher absorption in comparison to HbR. The absorption at 530 nm or 590 nm is insensitive to the oxygenated state of hemoglobin and is used to assess changes in hemoglobin concentration (HbT) [141]. State-of-the-art IOI uses standard charged coupled device (CCD) cameras to collect the reflected light, while the illumination source at a specific wavelength can be achieved with LEDs or filters applied to a white light source [141].

The first application of IOI in neuroscience was by Grinvald and colleagues [106] in 1986. In their work, IOI with light at a wavelength of 665 to 750 nm was used to map the activation of the exposed barrel cortex in anesthetized rats during mechanical stimulation of whiskers. In the same study, IOI was used to map orientational columns in the visual cortex of cats and monkeys, highlighting a potential implementation of IOS in cross-species research.

Over the years, IOI has been adapted for imaging in the entire cortex in rodents [142, 143]. White et al. [143] used IOI to study resting-state functional connectivity in the dorsal cortex in anesthetized mice with a FOV of 1 cm^2^ across the exposed intact skull. Specifically, they used multiple LEDs (478 nm, 588 nm, 610 nm, and 625 nm) to simultaneously acquire signals of both HbO_2_ and HbR and used these patterns for functional parcellation of the mouse cortex. Kura and colleagues [142] later compared the resting-state cortical connectivity maps based on IOS from multiple wavelengths versus those obtained from a single wavelength. The results revealed that connectivity maps based on IOS from HbO_2_ and HbR are quantitatively comparable with the maps based on HbT changes.

However, conventional IOI has some fundamental constraints. Since IOI is based on the hemodynamic response, it lacks cell type specificity and has limited temporal resolution compared with other optical methods. Furthermore, like all-optical imaging methods, it suffers from limitations in spatial resolution owing to signal scattering by the skull. In experiments where non-invasiveness is not a necessary parameter, IOI can be combined with other imaging techniques (e.g., calcium imaging), bypassing these limitations. For example, researchers from Hillman’s lab measured the IOS and GCaMP fluorescent signal from the bilaterally exposed dorsal cortex in awake, head-fixed mice [141]. The simultaneous assessment of IOS and fluorescent signals, named wide-field optical mapping (WFOM), allowed an increase in the spatial resolution after correcting for the cross-talk between the excitation and emission spectra of GCaMP and the absorption of oxygenated and deoxygenated hemoglobin. Ultimately, it was possible to achieve single-cell resolution, with cell type specificity ensured by the GCaMP expression.

#### Light scattering imaging

In the fluorescence imaging section, we have focused on practical approaches that rely on measurements of absorbed light. However, the interaction of light with the sample (i.e., the brain) is not limited to absorption and is also affected by scattering. In the following section, we will briefly discuss light scattered imaging. We limit our focus to two areas: (1) light scattering mechanisms and their influence on IOS and (2) laser speckle imaging.

##### Mechanisms of light scattering and its influence on IOS.

As summarized by Villinger and Chance [144], there are two types of light scattering associated with neural activity: fast and slow. In 1980 Tasaki, Iwasa, and Gibbons [145] described the physiological basis of fast scattering. They used a photon sensor located on the surface of a claw nerve to detect motion of the nerve surface upon traveling of an action potential in vitro. As shown later [144], this movement of the cell membrane leads to changes in the refractive index of the membrane, which ultimately affects the scattering of light. In the early 1990s MacVircar and Hochman studied the mechanism of slow scattering by measuring light transmission in the dendritic area of the CA1 region in slices of rat brain. Synaptic activity has been found to result in increased light transmission. This effect was related to the potential glial swelling triggered by the increased extracellular concentration of K+, which occurred during the generation of an action potential. The swelling of the cells causes less light scattering, therefore, increasing light transmission [146]. This could not be explained by changes in HbO2 absorbance [146, 147], thus exposing a mainly different factor that affects IOS.

The “fast” and “slow” scatterings were further confirmed by in vivo rodent experiments. Rector and colleagues [148] recorded IOS from the barrel cortex of anesthetized rats. Using a specifically designed fiber optic probe [149] that was placed on top of the dura matter, they measured fluctuations in scattered light intensity on a millisecond time scale upon twitching of the whisker. The data from “fast scattered” light were further used to map the individual columns in the barrel cortex. Subsequently, Pan et al. [150] were able to simultaneously measure two effects that drive IOS upon spontaneous neuronal activation: (1) the increased absorption driven by increased HbO_2_ concentration (2) the reduction of overall neural tissue scattering caused by neural tissue swelling. In brief, Pan and colleagues used two implantable optodes (i.e., a fiber pair): the light source and the detector, to measure the light transmission through the neural tissue of anesthetized rats. The pair of fibers were used to measure from either the primary somatosensory area or caudate-putamen. Importantly, both effects: absorption by HbO_2_ and scattering by swelled tissue, were on the same time scale—seconds, emphasizing the interference between slow scattering and IOS. This interference can lead to a potential misinterpretation of the imaging results. One of the possible solutions to circumvent this pitfall is the one suggested by Pan et al. simultaneous measurement of light absorption and scattering. Finally, the underlying physiology of slow scattering and fast scattering is independent of neurovascular coupling suggesting scattering as a separate imaging contrast [148, 150]. Despite the current limitation to a single area of the brain, the potential use of, for example, different pairs of fibers may allow for in vivo “imaging” from multiple brain areas.

##### Laser speckle imaging

In addition to the static scattering, in which particle motion is ignored, light scattering is also used for CBF quantification in Laser Speckle Contrast Imaging (LSCI). In short, if coherent light—such as from a laser—is scattered on moving particles—such as red blood cells—the resulting interference patterns, or speckles, will cause a dynamic change in the backscattered light.

In 1981, Ferchner and Briers [151] suggested making a short exposure (in the 10 ms interval [152]) of the speckle temporal fluctuations, thus converting the unknown distribution of the velocities to the variations of the speckle contrast. These contrast changes were then converted into intensity distributions, which reflect the velocity distribution of the moving particles. This approach established a basis for LSCI’s CBF visualization. In the early 2000s, Dunn and colleagues [152] were the first to use LSCI to measure CBF in anesthetized rats. The researchers used a CCD camera through the 6 × 6 mm FOV of the cortex of rats suffering from diffuse cortical depression or cerebral ischemia. The spatiotemporal resolution was 10 µm and 1 ms, respectively. In 2020, Postnov and colleagues [153] monitored CBF after stroke induction in mice with an exposure time of ~30 µs and a spatiotemporal resolution of 10 µm and 10 µs. Importantly, LSCI is currently used for clinical research in several fields of medicine, including neurology [154]. This increases the translational potential of this technique, which could in the near future lead to a general acceptance of LSCI as the standard for CBF monitoring in neurosurgery.

#### Photoacoustic imaging

Despite recent methodological breakthroughs, non-invasive in vivo optical microscopy still faces inherent optical limitations that restrict it to mostly cortical investigations [155]. To this end, photoacoustic (optoacoustic) imaging (PAI) offers high resolution in tissue depths far beyond current microscopy standards while maintaining rich optical contrast [156, 157]. In PAI, signal generation relies on the absorption of pulsed laser light at specific optical wavelengths by endogenous chromophores like oxy-/deoxy hemoglobin or exogenous CAs. Tissue heating owing to the process of photon absorption produces broadband acoustic waves at megahertz frequencies (photoacoustic effect) which can be detected at the tissue surface by ultrasound transducers and reconstructed based on the distribution of absorbed optical energy [156]. Although PAI’s prevailing application is in cancer research [158, 159], its non-invasive nature and ability to directly monitor biological processes make it an appealing tool for in vivo neuroimaging. The following part will review the most recent applications of PAI in wide-field functional imaging in rodents.

##### Photoacoustic visualization of in vivo neural dynamics

Given that PAI combines both optical excitation and acoustic detection, a variety of imaging techniques are available, with photoacoustic microscopy (PAM) and tomography (PAT) being the most promising ones for preclinical in vivo functional neuroimaging (see [160] for a comprehensive overview of PAI methods).

##### Photoacoustic microscopy

PAM operates by scanning a tightly focused laser beam point-by-point across the tissue surface and detecting the thermoelastically evoked acoustic waves [161]. It provides a comprehensive and quantitative characterization of cerebral hemodynamics with an excellent spatial resolution of a few microns [162]. An early application of PAM verified the “initial dip” of the BOLD fMRI hemodynamic response to an electric stimulus as rapid changes in arteriolar oxy-/deoxy hemoglobin ratios [163]. PAM systems have since been further developed and used for many applications in neuroscience [155, 164, 165]. Using acoustic-resolution PAM (AR-PAM), Stein et al. [166] non-invasively imaged blood-oxygenation dynamics of several cortical vessels in rodents during controlled hypoxia and hyperoxia challenges. Follow-up systems, capable of three-dimensional high-speed imaging, were introduced a few years after to non-invasively map cortical blood-oxygenation at the capillary level [161, 167, 168]. Modern PAM systems now enable the investigation of spontaneous cerebral hemodynamic fluctuations and their associated functional connections in rodent research [164, 169]. With contrast-enhancing agents like genetically encoded chromophores and voltage-sensitive dyes (VSD), the repertoire of PAI was extended to not only include the detection of hemodynamic processes but also direct detection and quantification of neuronal activity [170, 171]. For example, Shemetov et al. [172] engineered a genetically encoded calcium indicator with an increase of up to 600% in the fluorescence response to calcium. The probe was validated in vivo using hybrid photoacoustic and light-sheet microscopy, where both neuronal and hemodynamic activity could be captured with high resolution through the intact mouse skull. Despite constant technical advances, though, PAM’s high resolution and imaging speed are yet to be applicable to tissue penetration depths beyond 1 mm. In such cases, photoacoustic tomographic systems based on ring-shaped transducer arrays can provide deep brain volumetric visualization of hemodynamics and stimulus-evoked brain activity [155, 160, 173].

##### Photoacoustic tomography

PAT uses several wavelength lasers to evoke photoacoustic waves, making it possible to non-invasively and volumetrically measure varying concentrations of endogenous chromophores and exogenous CAs in deep brain tissue [174–176]. In fact, the first in vivo photoacoustic images of small animals were reconstructed based on PAT system scans from a rat’s head: Wang et al. [177] accurately mapped brain structures with and without lesions, as well as functional cerebral hemodynamic changes in cortical blood vessels around the barrel cortex in response to whisker stimulation. PAT has also been successfully applied to study whole-brain hemodynamics [176] and even resting-state functional connectivity in rodents [178]. In 2016, Tang et al. [179, 180] described a similar but wearable cap-like PAT system for awake and behaving rats, with a high in-plane spatial resolution of 200 µm at depths of up to 11 mm. In a mouse model of epilepsy, oxy-/deoxy hemoglobin-based photoacoustic computed tomography (PACT) scanning system captured the superficial epileptic wave spreading around the epileptic focus and a corresponding wave propagating in the opposite hemisphere [181]. Going beyond superficial cortical measurements, a newly devised system combining PACT and electrophysiological recordings enabled the first non-invasive visualization of real-time thalamocortical activity during an epileptic seizure in the whole mouse brain. Furthermore, endogenous contrast-based PACT successfully mapped brain-wide activation during electrical fore- and hind-paw stimulation in mice to their somatosensory cortex forelimb and hindlimb areas, respectively [182]. Using contrast enhancement, Gottschalk et al. [183] recently measured real-time in vivo calcium transients across the mouse brain by devising a functional PA neuro-tomography setup. They reached sufficient sensitivity to directly detect fast neural responses to electrical hind-paw stimulation. Using a near-infrared VSD, Kang et al. [184] monitored in vivo chemically evoked seizures in rats at sub-mm spatial resolution, without the need for invasive craniotomy of skull thinning.

Although still in its infancy, whole-brain functional PAI has rapidly evolved in recent years to meet the standards of the mainstay neuroimaging methods. With its multiscale imaging capabilities comprising rich optical contrast, high spatial resolution, and imaging rate, PAI sits at a unique position to directly visualize key parameters of brain function. Although PAI does come with its own set of limitations, much like any other neuroimaging technique, it nevertheless shows great potential to bridge the gap between micro- and macroscopic functional neuroimaging.

#### Alternative methods for whole-brain functional mapping and data analysis

In this review, we focused on a group of rapidly developing imaging methods that allow us to study the interactions between groups of neurons in a network. However, there are other ways to study brain function, which will be mentioned here. A technique that has existed since the first half of the twentieth century is electroencephalography on rodents [185]. Electroencephalography enables high-resolution visualization of brain activity but with very limited spatial coverage. It is mostly utilized to study brain oscillation patterns in sleep, epilepsy, schizophrenia, or as a biomarker of pharmacological activity of centrally active drugs [185, 186]. The recent developments in rodent electroencephalography, along with translational benefits and pitfalls of the technique regarding the study of the rodent brain in health and disease have recently been outlined in detailed reviews (see [185–189]).

An additional method that allows for the evaluation of behavior-induced neural activity in rodents is based on the use of genes regulated by neuronal activity, such as Fos. Fos is a transcription factor induced by neural activity resulting from emotional arousal or sensory signals [190, 191]. Immunostaining of this immediate-early transcription factor allowed scientists to generate whole-brain maps of behavior-induced neural activity in rodents [191–193]. In one of the first applications of this approach, Wheeler and colleagues [191] studied brain-level maps of memory recall-induced expression of Fos, which was characterized through a fear conditioning task. Using graph theory analysis of regional Fos quantification to study changes in network connectivity as a function of memory recall, they found that memory recall involves activation of distributed network sets, with hubs in the prefrontal cortex and in the thalamus. Multiple comprehensive maps of Fos expression were generated to study brain activity induced by a number of different behaviors such as fear [194], fear learning, and recall [190] as well as alcohol addiction and withdrawal [193]. Evaluation of Fos expression maps at the brain level helps in the mapping of cellular networks involved in the expression of normal behaviors and in the in-depth investigation of circuit dysfunction in mouse models of neurological diseases.

Constant developments and advances in micro- and mesoscale imaging methods have prompted the creation of new tools for evaluating the function of whole-brain circuits. This has led to a series of interactive computational structures that automatically annotate, analyze, visualize and share whole-brain data at cellular resolution, using the interactive mouse brain atlas. Specifically, Furth et al. [195] developed an open-source software solution “WholeBrain”, which enables the quantification and spatial mapping of multidimensional data from whole-brain experiments, followed by “Openbrainmap” which enables data visualization and sharing in an interactive web-based framework. Another computational interface for single-resolution whole mouse brain analysis is CUBIC-Cloud [196]. CUBIC-Cloud builds the user’s mouse brain database from the 3D image stack, whereas the graphical user interface tools perform various types of quantification tasks. There are numerous other frameworks that allow a solid reconstruction and recording of the whole mouse brain, such as “ClearMap” [197], “BrainsMapi” [198], “NeuroInfo” [199], to name but a few. The availability of these open-source computational frameworks not only simplifies the analysis of large imaging data sets but also facilitates the rapid comparison and sharing of data between projects and laboratories.

## Conclusions

Until recently, the inability to measure brain activity in its entirety and simultaneously link it to physiologically relevant processes has limited our understanding of the relationship between neuronal activity and complex cognitive processes. This has changed dramatically with new large-scale functional imaging methods, which allow us to study previously invisible processes both in real-time and longitudinally, and to associate these processes with healthy or impaired neural function.

In this review, we have seen how the development of these approaches has allowed for higher spatial resolution, a wider FOV, and greater temporal resolution. However, there are still many limitations of these techniques that prevent a full understanding of local and global brain dynamics and their impact on behavior and cognition. We have outlined some of these gaps in each section of this review to guide current and future investigators (see Table 1).

**Table 1:** Table summarizing the costs, the major technical difficulties, the translatability, and the type of restraining method of each technique.

Imaging Technique	Equipment Cost	Translatability	Rodent restrain method
**Preclinical Magnetic Resonance Imaging**	high purchase costs medium operating and maintenance costs	possible	awake and anesthetized, head-fixed animals
**Ultrasound imaging**	low to medium purchase costs low operating and maintenance costs	possible	awake and behaving, freely moving animals
**Widefield, Confocal & Multiphoton**	medium purchase costs low operating and maintenance costs	not possible	awake and anesthetized, head-fixed animals behaving, head-fixed animals
**Intrinsic Optical Imaging**	low purchase costs low operating and maintenance costs	potential exists	awake and anesthetized, head-fixed animals behaving, head-fixed animals
**Light scattering imaging**	low purchase costs low operating and maintenance costs	potential exists	awake and anesthetized, head-fixed animals behaving, head-fixed animals
**Photoacousting imaging**	medium purchase costs low operating and maintenance costs	potential exists	awake and anesthetized, head-fixed animals behaving, head-fixed animals

High cost >100,000; *mediumcost* = 50,000–100,000; low cost <50,000$.

Besides the precise measurement of neural activity, the field of functional imaging also requires integration with computational models capable of using this data in order to make predictions about behavioral functions, which can then be tested experimentally. However, this has not been addressed here to give greater emphasis to the technical/experimental aspect of the methods discussed. The challenge for the future is therefore not only to design and engineer systems capable of detecting brain functions with the highest possible precision and accuracy, but also to integrate and analyze this data within theoretical models to develop a complete picture that can guide our understanding of the human brain. This review aims to attract new researchers to help unravel the mysteries of large-scale neural activity.
